# Investigation of the North Face Corridor in the Great Pyramid of Giza using Electrical Resistivity Tomography

**DOI:** 10.1038/s41598-025-29081-4

**Published:** 2025-11-21

**Authors:** Polina Pugacheva, Hussien Allam, Mohamed Fath-Elbab, Mohamed Sholqamy, Khaled Taie, Khalid Helal, Olga Popovych, Mohamed Elkarmoty, Norbert Klitzsch, Mehdi Tayoubi, Hany Helal, Christian U. Grosse

**Affiliations:** 1 Chair of Non-destructive Testing, TUM School of Engineering and Design, Technical University of Munich, Lichtenbergstrasse 2, 85748 Garching near Munich, Germany; 2 Rock Engineering Laboratory, Faculty of Engineering, Cairo University, Gamaa Street 1, Giza, 12613 Egypt; 3 UNESCO Chair on Science and Technology for Cultural Heritage, Faculty of Engineering, Cairo University, Gamaa Street 1, Giza, 12613 Egypt; 4 Department of Mining, Petroleum, and Metallurgical Engineering, Faculty of Engineering, Cairo University, Gamaa Street 1, Giza, 12613 Egypt; 5 Chair of Computational Geoscience, Geothermics and Reservoir Geophysics, RWTH Aachen University, Mathieustr. 30, 52074 Aachen, Germany; 6 Dassault Systèmes, 10 Rue Marcel Dassault, 78140 Vélizy-Villacoublay, France; 7 Heritage Innovation Preservation Institute (HIP Institute), 50 Rue de Rome, 75008 Paris, France

**Keywords:** Electrical Resistivity Tomography, 3D CAD model, Great Pyramid of Giza, Chevron, North Face Corridor, ScanPyramids, Engineering, Physics, Solid Earth sciences

## Abstract

Despite some impressive examples of Electrical Resistivity Tomography (ERT) characterizing the internal structure of historical monuments, ERT is rarely considered a primary method for this purpose because it is challenging to adjust measurement procedures and inversion techniques for such intricate objects. In this study, ERT was first applied in the Great Pyramid of Giza to detect the presence of the ScanPyramids North Face Corridor (SP-NFC). The ERT measurement technique and data analysis procedure were adapted for this case, which was characterized by complex surface topography and limited space for placing ERT lines. A 3D CAD model of the Chevron area was designed for inversion based on a 3D point cloud. The inversion results show the existence of the SP-NFC, with average dimensions of approximately 2.5 m by 2.5 m, starting at a depth of around 1 m and extending at least 2 m into the pyramid. The ERT study thus provided volumetric data confirming both the size and extent of the SP-NFC, complementing the ground-penetrating radar (GPR) and ultrasound tomography (UST) studies conducted simultaneously in the Chevron area.

## Introduction

### Background and objectives

**Fig. 1:**
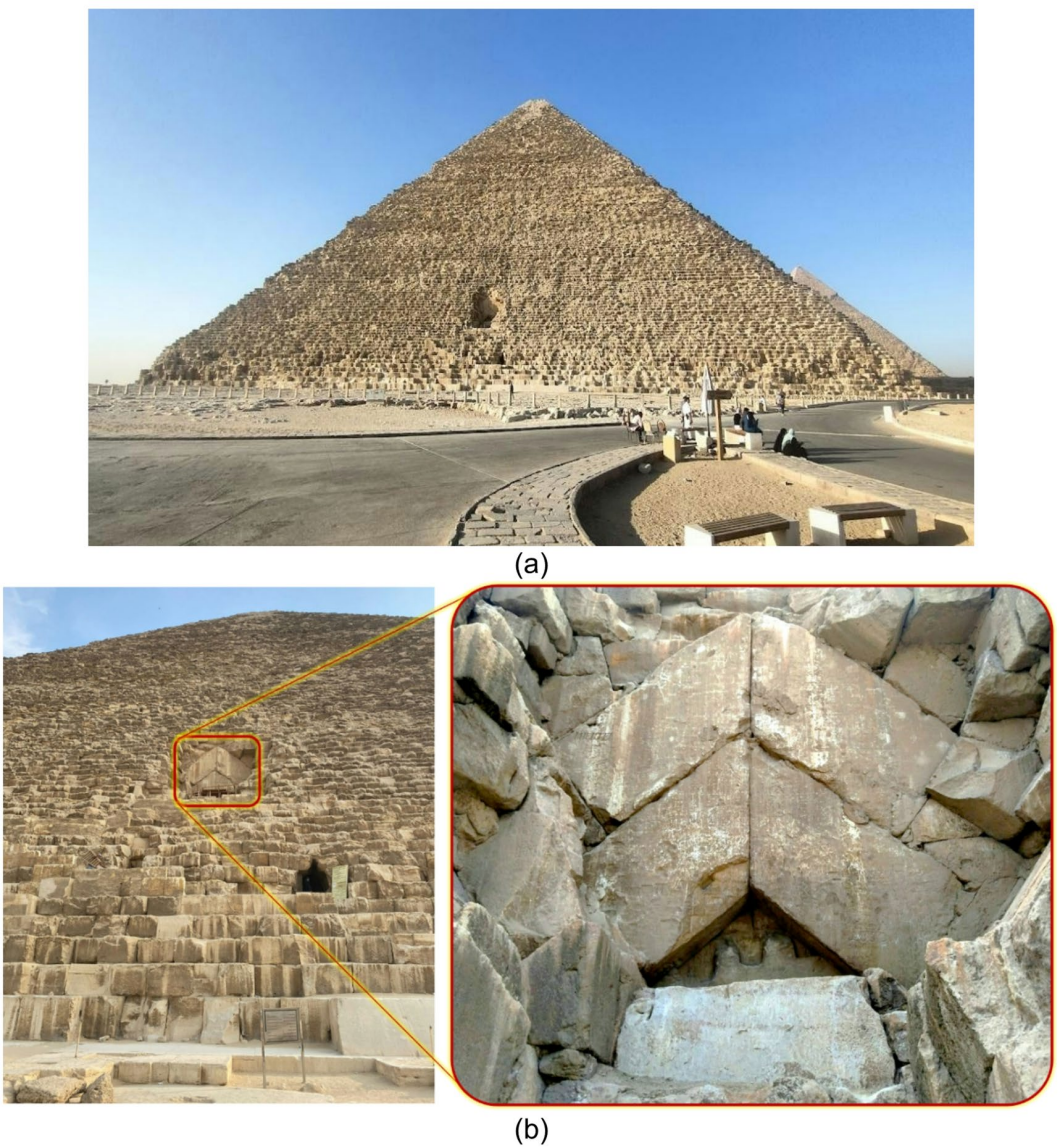
(**a**) Photo of the Great Pyramid of Giza, and (**b**) Photo of the Great Pyramid’s Northern face (left) and the Chevron area (right).

A recognized symbol of ancient Egyptian civilization, the Great Pyramid of Giza (Fig. 1a) was built with exceptional precision for its time. One of the main keys to understanding the technology of its construction lies in the pyramid’s internal structure; however, a significant part of it remains insufficiently studied. In this study, Electrical Resistivity Tomography (ERT) was used to investigate the Great Pyramid’s internal structure in the Chevron area (further Chevron).

In the last decade, scientists from the international research project ScanPyramids have made significant progress in studying the Great Pyramid through multidimensional muon radiography^1,2^. The idea of using cosmic ray muons to explore the Egyptian pyramids was proposed by Luis Alvarez several decades ago^3^. Further development was achieved through the introduction of modern measuring instruments and sophisticated reconstruction algorithms that make it possible to identify areas of anomalously abundant accumulation of muons within the pyramid^4^. The project’s key findings include the detection of two voids, a large one situated above the Grand Gallery and a smaller corridor on the pyramid’s North face^2,5^. The existence of such a corridor above the main entrance was predicted by French architect Jean-Pierre Houdin^6^. According to his hypothesis, the corridor was the initial section of the ‘Noble Circuit’ and connected the King’s and Queen’s Chambers at the heart of the monument. An endoscopic survey of the SP-NFC was conducted in 2023, shortly after its exact location was determined using GPR and ultrasonic testing^7^. The survey revealed that the SP-NFC features a high vaulted ceiling and a relatively large width, which is unusual for the Great Pyramid corridors except for the Grand Gallery. Following the confirmation of the SP-NFC, some of the findings were further analyzed in detail using the image fusion technique^8^.

Electrical resistivity tomography is a well-established geophysical method for detecting air-filled voids and tunnels. Its effectiveness is based on the large differences in resistivity between air and the surrounding materials. However, the success of such investigations depends on several factors, including the depth and size of the void, the nature of the host materials, and the suitability of the survey methodology and data analysis for the specific site conditions^9–14^. By employing flat electrodes, measurements can be carried out on marble, limestone, and chalk surfaces, materials traditionally considered unsuitable for ERT due to their low conductivity^15–18^. In 1977, Moussa, Dolphin, and Mokhtar successfully carried out resistivity measurements using flat electrodes coupled to the rock with a stiff mud slurry in the corridor of the Pyramid of Khafre^16^. Their primary aim was to detect shafts, tombs, and tunnels within the limestone bedrock surrounding the major pyramids and the Sphinx. In most cases, the researchers acquired high-quality data and identified anomalies; however, at one location near the Great Pyramid, the data was considered unreliable due to low current injected into the ground.

The object of an ERT study can be a building’s single structural element, such as a wall, floor, and ceiling, or an entire structure modeled in closed three-dimensional geometry. Tejero-Andrade et al.^18^ and Chavez et al.^19^ effectively used the latter approach to reconstruct in 3D the internal structure of the ancient Pyramid of El Castillo. Taking advantage of the Mayan stepped pyramid shape, the authors used an ensemble of unconventional ‘L’ and ‘Corner’ electrode arrays deployed around the pyramid steps and base in a square geometry to explore its interior and foundation, and discovered several previously unknown underground structures, including a buried cavity, partially filled with water^18,19^.

The Great Pyramid, one of the largest historical monuments in the world, is a complex subject for ERT research at all stages, from data collection to inversion and interpretation. The strengths of ERT cannot be fully exploited at the Great Pyramid because most of its outer area cannot be reached safely, and, as a result, it is impossible to conduct measurements there. The only exception is the Chevron area, accessible via a temporary scaffolding construction. The Chevron is located in a niche on the Northern face of the Great Pyramid, where the relief is most pronounced, with height differences between the stone ledges and depressions exceeding three meters (Fig. 1b). This significantly complicates not only ERT measurements but also data processing due to the limitations of inversion software when dealing with complex topographic features.

At the Chevron, the need to work along the vertical walls of the pyramid imposed additional design requirements for the electrodes; they had to be flexible enough to adapt to the uneven surface of the rock, and light enough to be mounted on a vertical surface. In this context, so-called mesh electrodes offer a good alternative to traditional plate electrodes. Currently, various types of mesh electrodes are widely used for applications requiring lightweight, non-invasive electrodes, particularly in medical diagnostics and chemical engineering. In geophysical applications, mesh electrodes may be a potential solution to the problem of high contact impedance in regions with resistive surface materials, such as permafrost. For instance, Tomaskovicova et al. (2016) demonstrated that mesh electrodes have 29–37% lower contact resistance in frozen soil than plate electrodes of similar size^20^.

This study’s objective is to investigate the applicability of the ERT method for detecting the ScanPyramids North Face Corridor (SP-NFC) in the Chevron area of ​​the Great Pyramid, which is characterized by complex relief. ERT measurements were conducted in three field campaigns, from 2020 to 2022, at the Chevron site (Fig. 1a), located above the entrance tunnel. To properly account for the three-dimensional effects of the Chevron’s topography and the internal structures of the pyramid, the measurement data was processed using a 3D inversion algorithm.

This article begins by explaining the basic principles of the ERT method, followed by describing data acquisition and 3D modeling strategy proposed for this ERT case. Next, in Sect. 3, the results of the inversion of synthetic and field data are presented and then compared with each other as well as with GPR, UST, and muon radiography measurement data in Sect. 4. Finally, the challenges and limitations of adopting ERT for investigating the Great Pyramid are discussed.

### Study site

The Great Pyramid is the central element of the Pyramids complex located on the Nile River’s west bank, which also includes the Khafre and Menkaure pyramids, the Sphinx, and the small Queens pyramids. One of the distinctive features of the Great Pyramid is the so-called “Entrance Vault,” located on the Northern face around 7 m above the entrance. It contrasts with the rest of the masonry by the unusual arrangement of blocks in an inverted V-shaped (Chevron) form. The stone vault protects the pyramid’s entrance from the gravitational load produced by the masonry above it^21^. The chevrons visible today were originally hidden under several layers of stone blocks, arranged exactly as expected from the Chevron block abutments on both sides of the monument. A triangular platform resembling a tympanum is directly below the Chevron arch. An asymmetrical lintel block partially overlaps it at the front. The distinctive ‘jagged’ relief of this block suggests that it may conceal an entrance and can be pushed aside with a lever.

## Methods

### Principles of electrical resistivity tomography

The ERT method is categorized as one of the electrical active source methods. ERT uses direct or alternating currents of very low frequency, i.e., predominantly conduction currents, and is directly sensitive to electrical resistivity (*ρ*), which defines the potential of a material to oppose current flow.

The most commonly used four-point measurement system consists of two electrode pairs acting as current and potential dipoles. The measurement gives the resistance, R, according to Ohm’s law, which is further converted into apparent resistivity by multiplying the geometric factor. This constant depends on the spatial arrangement of electrodes^22^. The apparent resistivity is equal to true resistivity only in the case of a perfectly homogeneous and isotropic earth (half-space); if that is not the case, then a single apparent resistivity value contains information about resistivity distribution for the whole medium’s volume, where the current propagates. However, different parts of this volume contribute unequally to the overall response, depending on the spatial sensitivity of the electrode configuration. In resistivity inversion, the sensitivity function, derived from the Jacobian matrix, is one of the main modules that links the measured data to the subsurface resistivity distribution and governs the iterative updating of the model parameters^23^. The sum of the absolute sensitivities of all quadrupoles in an ERT survey gives the coverage, also known as the cumulative spatial sensitivity. It indicates how well the measurements “illuminate” or constrain each model cell in the subsurface.

In studies aimed at detecting geological or anthropogenic structures of limited size and complex geometric shapes such as cavities and tunnels, the 3D inversion gives better results compared to 2D inversion^11,24^. The reason is that the 2D inversion algorithm calculates the electrical potential distribution in two-dimensional space, although the measurement data contains resistivity information across the entire volume of the medium through which the current propagates. This simplification does not distort the results of 2D ERT inversion if the lateral resistivity variations adjacent to the survey line are small relative to the other dimensions. In more complex environments, 2D analysis becomes insufficient, as resistive or conductive structures located close to the profile, though out of plane, may be projected onto the 2D ERT section^25,26^.

There are two main approaches for obtaining ERT data that enable 3D data analysis. The first is to use a true 3D measurement setup with electrodes arranged uniformly in a rectangular or circular grid or, if possible, at different depths. With such electrode placement, measurements can be made for different combinations of current and potential electrodes in many directions. An alternative is to measure the data in 2D survey geometry, i.e., along several parallel profiles in one or two perpendicular directions and combine them into one data set for 3D inversion^22^. With small distances between adjacent lines, ideally no more than one or two electrode spacings, this approach can provide good geometry reconstruction of complex 3D structures^22,27,28^.

### ERT data acquisition

**Fig. 2:**
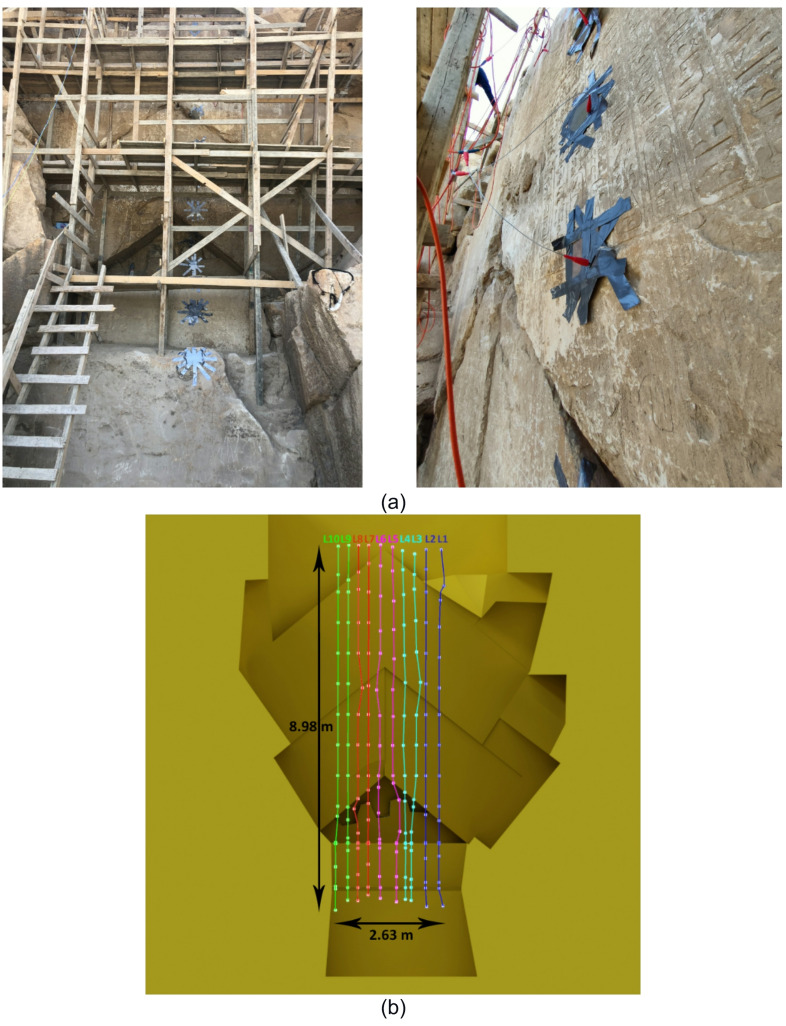
(**a**) ERT measurement setup in the Chevron area using mesh electrodes, and (**b**) Layout of all measured 2D ERT lines (L1–L10) plotted on the simplified 3D model.

The measurements were carried out on ten vertical quasi-parallel lines with a distance between lines of 0.25 m and electrode spacing of 0.75 m (Fig. 2b). Due to the direct relation between the total ERT line length and the depth of investigation, it was necessary to make the lines as long as possible, including areas with high variations in topography.

The dipole–dipole (DD) configuration was chosen for this study because of its high sensitivity to lateral resistivity variations, enabling more accurate reconstruction of complex structures, such as cavities and tunnels, compared to alternative Wenner or Schlumberger configurations^22,29^.

The measurements were conducted using the Terrameter LS2 device manufactured by ABEM. In the measurement protocol, the electrode spacing (a) ranged from 2 to 5, while the dipole separation factors (n) varied from 1 to 5. For this application, specially designed flat stainless steel woven wire mesh electrodes (0.20 m × 0.20 m) were securely attached to the pyramid’s wall (Fig. 2a). A synthetic sponge soaked in fresh water was placed between the mesh electrodes and the stone to ensure good galvanic coupling.

Data quality was assessed based on stacking and reciprocity errors. Due to time constraints during the surveys, reciprocal measurements were collected for a reduced data set only. The final data set was processed to exclude data points with distinct outliers and negative apparent resistivity values. Additionally, all data points collected from each line’s first two electrodes were excluded due to poor contact between the electrodes and the highly uneven surface of the upper limestone blocks, which hindered reliable galvanic coupling. Before the 3D inversion, preliminary 2D ERT analyses were conducted on individual resistivity profiles. In the next step, all collected data, and the X, Y, and Z coordinates of the electrode positions were combined into a single data set and inverted using a 3D inversion algorithm to properly account for the effects of the pyramid geometry, including its internal voids and Chevron relief.

**Fig. 3:**
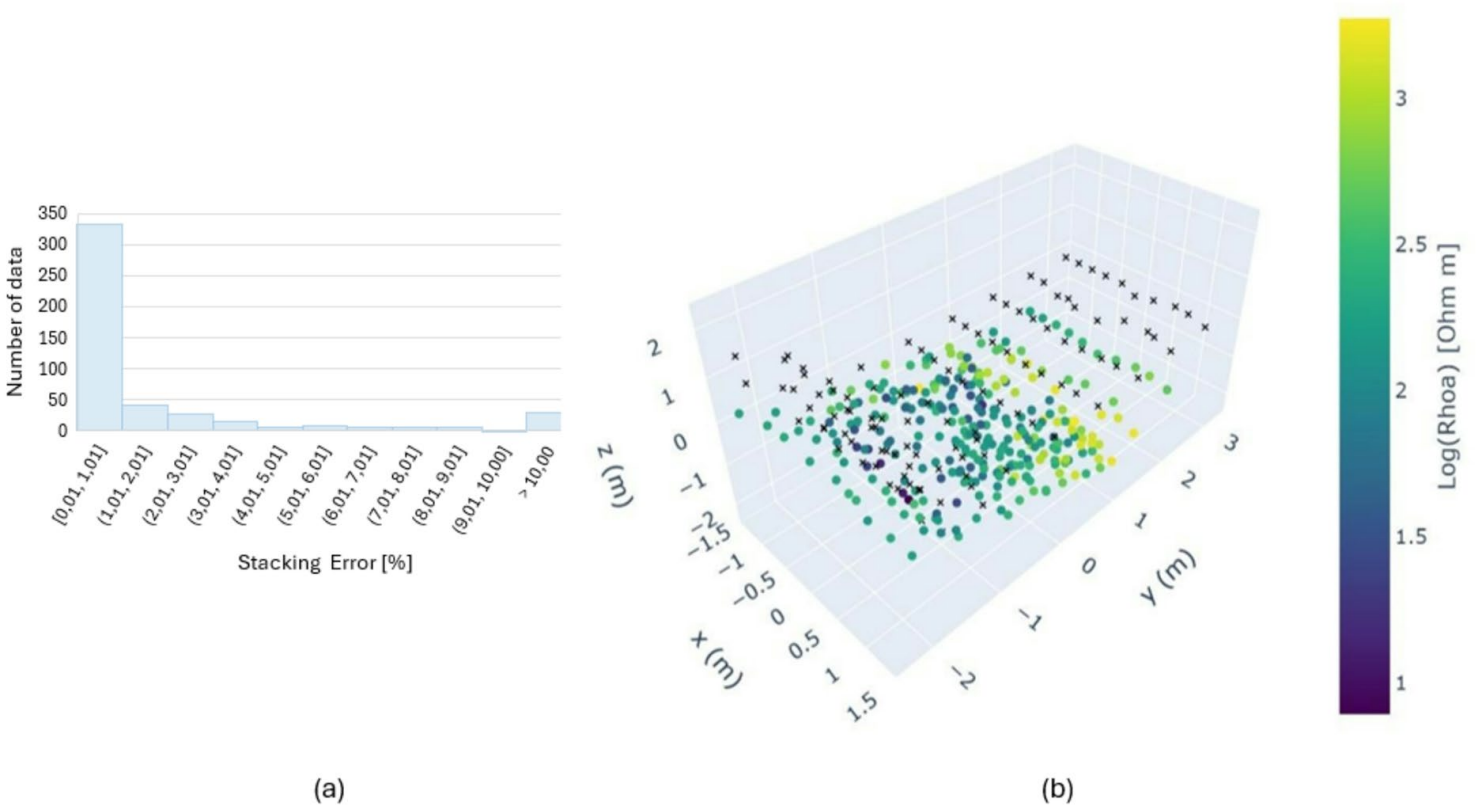
(**a**) Histogram of ERT measurement stacking errors [%], (**b**) The 3D pseudo-section illustrates the data points used in the 3D data inversion. Black crosses mark the electrode positions, and colored dots represent the apparent resistivities plotted at the midpoints of four electrodes involved in the measurement. Positive Y-coordinates correspond to the upper part of the Chevron (north), while positive X-coordinates denote the right side of the Chevron. The position of the plotting point along the Z axis (pseudo-depth) for each dipole–dipole measurement was estimated following Edwards (1977) as the median depth of investigation, determined by considering the electrode spacing and the dipole separation factor^30^.

The data was collected with varying current strengths, ranging from 36.7 mA to 199.1 mA, with a mean value of 134 mA. The mean contact resistance value calculated for all ERT lines was 1.7 kΩ. Analysis of measurement errors showed low data variability, with a mean stacking error of 0.2% and most stacking error values remaining below 1% (Fig. 3a). Reciprocal errors across different profiles ranged from 2% to 8%, with a mean of 4% for all lines. In the pseudo-section (Fig. 3b), the apparent resistivity data exhibited a relatively uniform distribution, with predominant values in the 150–350 Ωm range. In the central part of the pseudo-section, a distinct zone of high resistivity values (> 1000 Ωm, in yellow) was visible. Apparent resistivity values below 150 Ωm were observed in the lower part of the Chevron, between Y = 0 m and Y = -1 m, close to the surface.

### 3D CAD model of the Chevron area

**Fig. 4:**
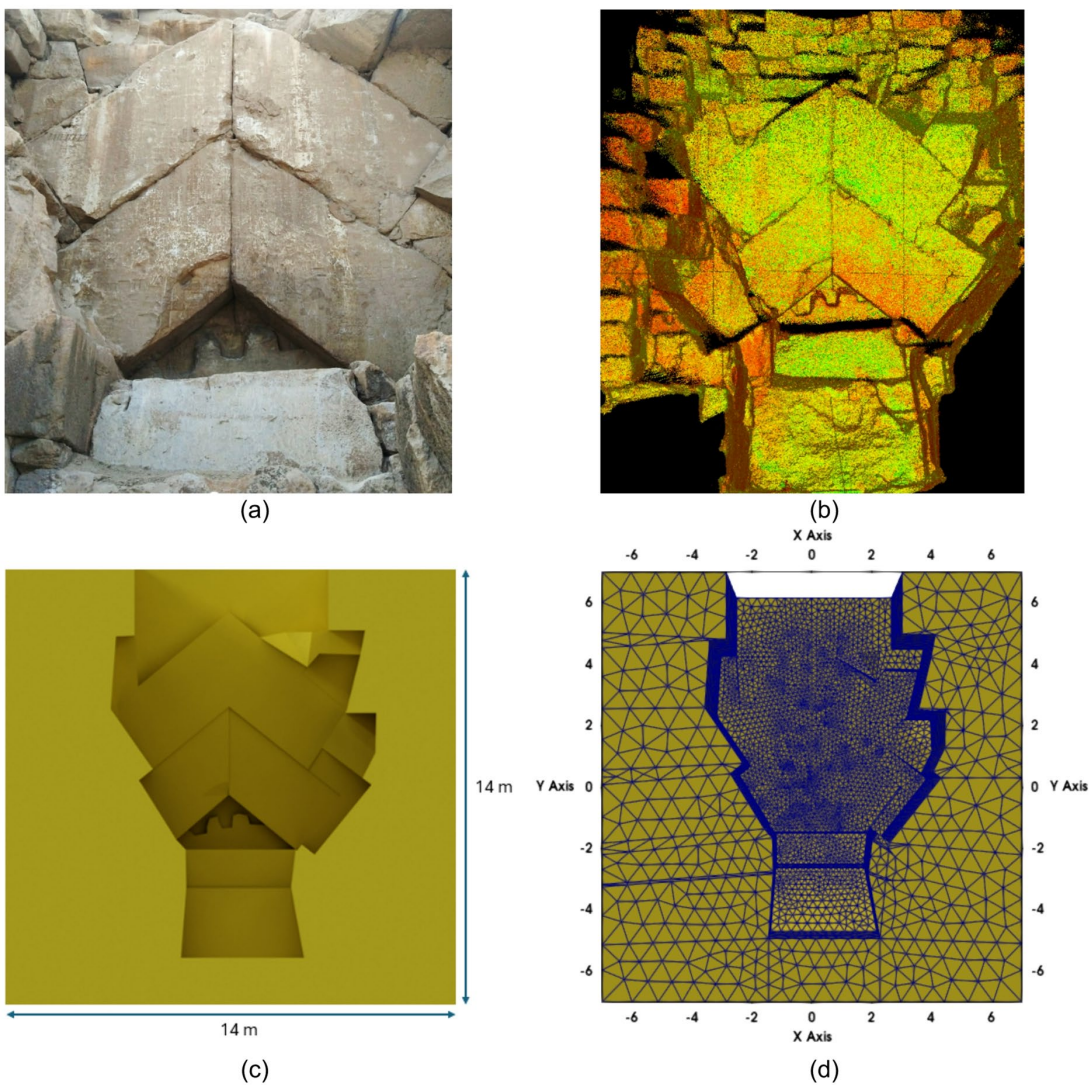
(**a**) Photo of the Chevron area of the Great Pyramid, (**b**) 3D point cloud after adjusting in Autodesk ReCap©, (**c**) 3D inner model designed using Autodesk AutoCAD© and then reconstructed as a solid body in Autodesk Inventor©, with final dimensions of 14 m × 14 m × 9 m; and (**d**) the finalized comp solid 3D model in Paraview© after meshing.

The electrodes’ topography points alone were insufficient to accurately reconstruct the surface topography in a three-dimensional model of the Chevron area (Fig. 4a). To solve this, high-resolution point cloud data obtained from a Zeb-Horizon 3D laser scanner (equipped with a VLP-16 sensor) was used for a detailed 3D reconstruction of the actual geometry, with the help of surveying control points collected by a total station device. However, the intricate surface shape derived from laser scanning data cannot be directly converted into a 3D model, as this would require a very fine mesh. Therefore, the Chevron geometry in the 3D model was simplified, while the main features of the relief were preserved.

The 3D point cloud data was adjusted and cropped using Autodesk ReCap© software to fit the specific surveying system and a chosen origin point on the Chevron (Fig. 4b). The adjusted 3D point cloud and the surveying control points were then imported to Autodesk AutoCAD© software and used as a guide to design the lines and surfaces of the simplified model, as closely as possible to the real case (Fig. 4c). After creating the surfaces of the 3D model, they were imported into Autodesk Inventor© for revision and reconstruction as a 3D solid body (inner and outer models). Next, they were combined into one comp-solid with FreeCAD software and then meshed concerning electrode positions using Gmsh (Fig. 4d)^31^. For such a complex 3D model, the unstructured tetrahedral mesh type was chosen, allowing more flexibility and better discretization than other mesh types^32,33^.

### Forward and inverse ERT modeling

**Fig. 5:**
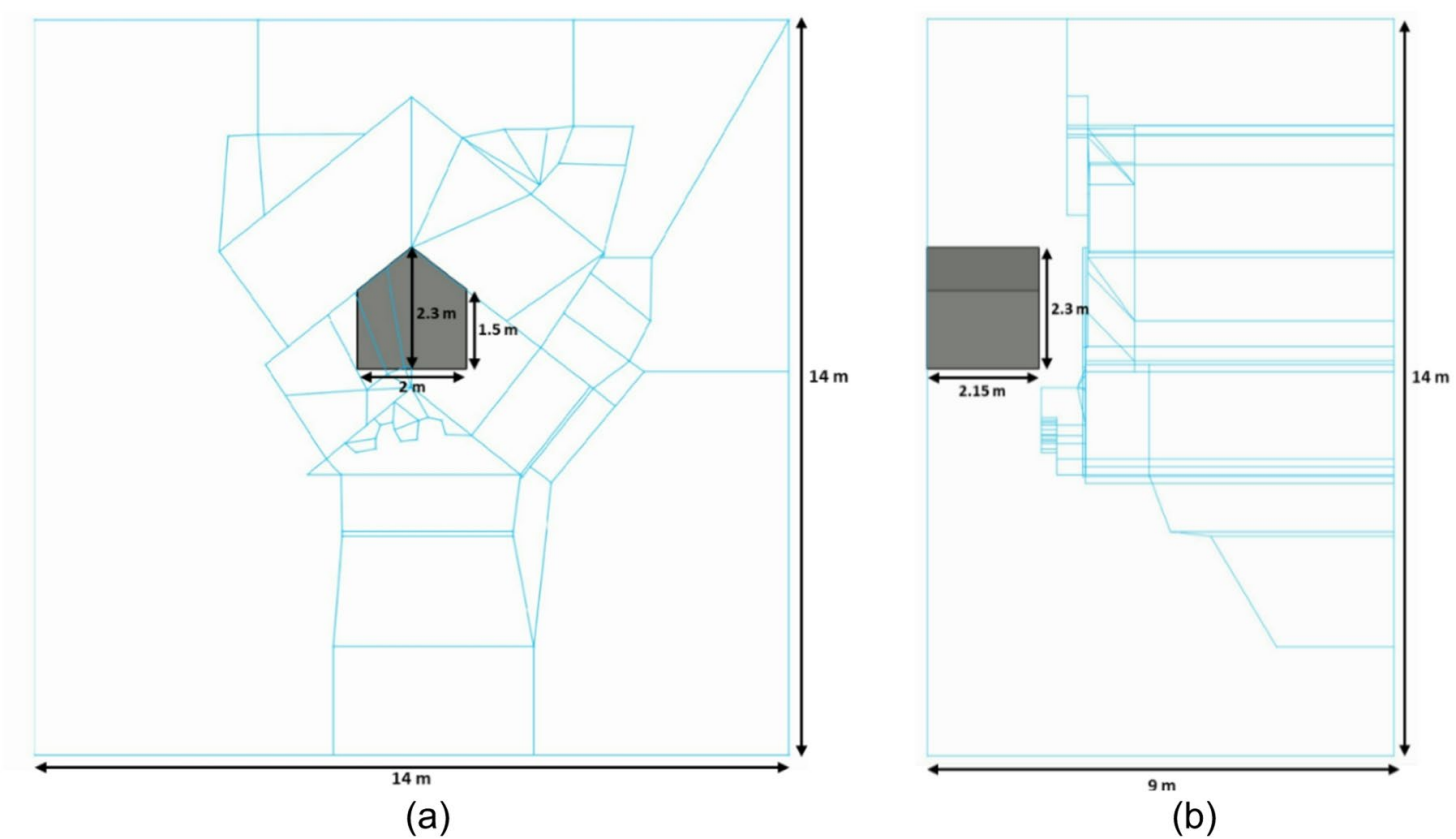
3D ERT model of the Chevron area with the air-filled corridor (the SP-NFC is shown in grey, and the boundaries of the inner model are highlighted in blue): (**a**) front view, (**b**) side view.

For forward modelling and inversion of ERT data, the open-source library pyGIMLi^34^ was used. The 3D synthetic modeling was conducted to evaluate the ERT method’s ability to image SP-NFC and to validate the results of the field data inversion. Inversion of the synthetic data set should show how accurately the position of the SP-NFC and its dimensions can be reconstructed using inversion parameters and DD configuration arrays identical to those in the field campaign. Based on positional and geometric data obtained from GPR measurements^7^, a synthetic 3D model was created, representing a 2-m wide air-filled corridor with a resistivity of$\:1.0$ × $\:{10}^{6}$Ωm, topped by a triangular vaulted ceiling reaching a maximum height of 2.3 m. The corridor begins at a depth of 0.85 m and extends inward to 3 m, as shown in Fig. 5a-b. In this 3D simulation, the focus was placed on a scenario with a homogeneous background resistivity of 500 Ωm. To model a multi-component material, a simplified approach was adopted, in which the limestone masonry is considered as a homogeneous medium, neglecting the mortar-filled joints between the stone blocks.

The parameters used for forward modeling are listed in Table 1. The inversion parameters and the relative and absolute voltage errors were identical to those used for the inversion of the field data. We set Neumann boundary conditions on the Chevron surface to prevent current flow perpendicular to the surface, and (mixed) Robin boundary conditions at all other surfaces to allow interactions with the surrounding media.

**Table 1:** Forward modeling parameters.

Forward modeling parameters	
Parameter	Value
Array	Dipole-dipole
Noise level	4%
Voltage dependent error	0.00002 V
Resistivity of limestone	500 Ωm
Resistivity of air	1,000,000 Ωm

The field measurement data was initially inverted in 2D mode on a triangular mesh with two nodes between adjacent electrodes. Such fine mesh is necessary to model the surface geometry accurately, since rough topography, affecting current propagation, may interfere with SP-NFC detection or cause false anomalies in the inversion results. In the following step, the data was combined into a single dataset. A 4% noise was added to account for measurement reciprocity errors, along with a voltage-dependent error of 20 µV to represent uncertainty in the measured potential. Together, these errors define the data error model used in the inversion procedure and determine the weighting of each data point in the objective function. The field data was inverted using a 3D inversion algorithm based on the L1 norm with low regularization (λ = 1) to reproduce the strong contrast between the air-filled void and limestone host medium on the Chevron. A weighting factor of 1 for isotropic smoothing was chosen to avoid introducing any assumptions about the predominant direction of resistivity changes.

## Results

### 3D inversion results of synthetic data

**Fig. 6:**
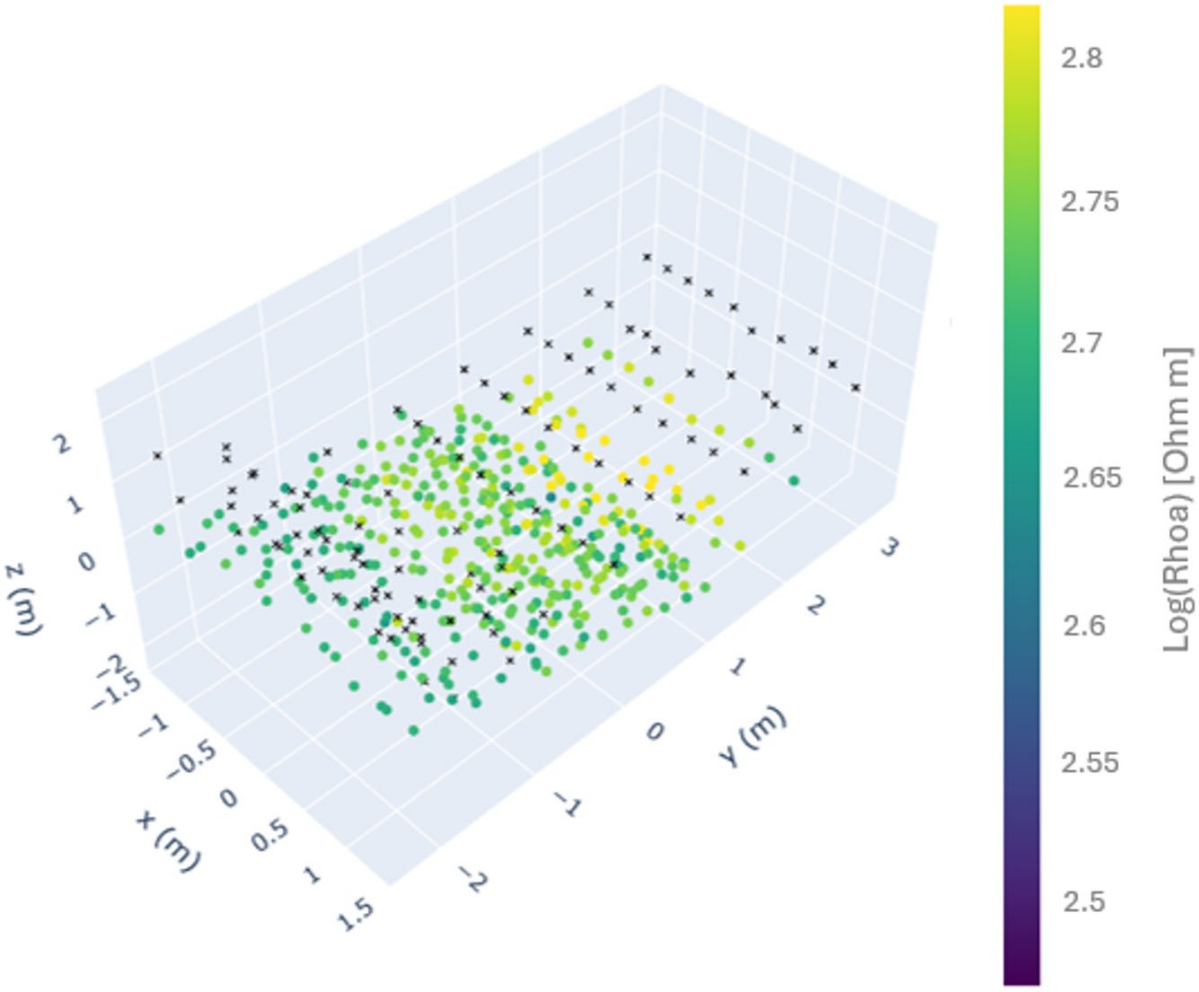
The 3D pseudo-section of the synthetic data. Black crosses mark the electrode positions, and colored dots represent the apparent resistivities plotted at the midpoints of the four electrodes involved in the measurement. Positive Y-coordinates correspond to the upper part of the Chevron (north), while positive X-coordinates denote the right side of the Chevron. The z-value (pseudo-depth) was estimated following Edwards (1977)^30^.

Figure 6 shows the 3-D pseudo-section of the forward model. Most apparent resistivity values range from 400 to 500 Ωm, indicating a relatively homogeneous distribution, whereas the area corresponding to the corridor exhibits higher apparent resistivity values of approximately 600 to 700 Ωm.

**Fig. 7:**
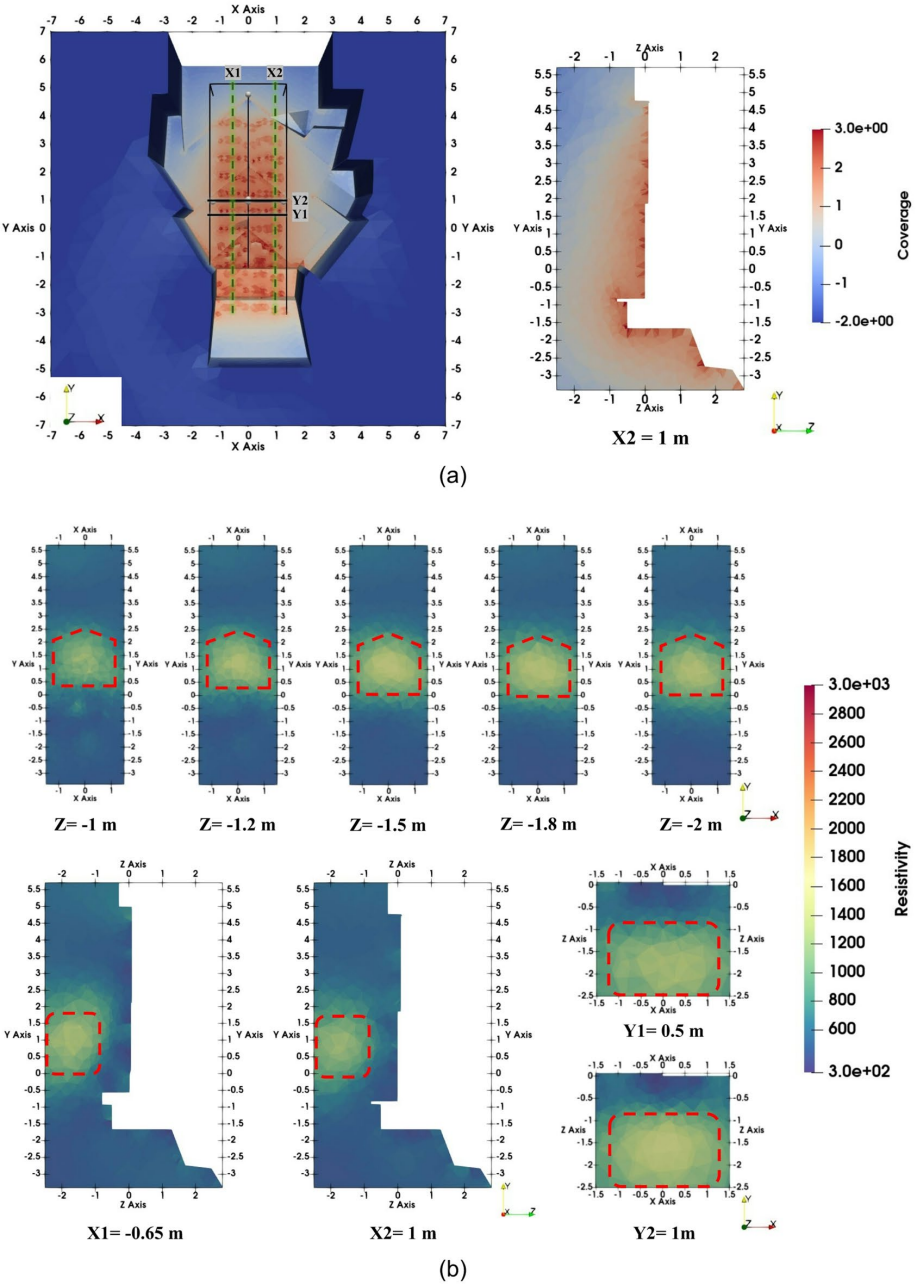
3D inversion results of the synthetic data: (**a**) coverage of the entire model with the location of X and Y slices on the cropped part of the model (black box) and coverage of slice X2; and (**b**) X-, Y-, and Z-slices of the inverted resistivity distribution.

All inversion results were visualized using Paraview^35^ and presented on a linear color scale to emphasize resistivity contrasts. Figure 7a illustrates the entire model’s coverage, indicating the locations of the X and Y slices on the cropped part (depicted by the black box) along with the coverage of slice X2 at 1 m. The inversion stopped after two iterations. The model obtained fits the synthetic data with an RMS error of 3%, which is close to the input noise level. The coverage distribution indicates that model parameters can be well resolved to a depth of 1.5 m, and satisfactorily extend to 2 m. Below Z = − 2 m and in the upper part of the Chevron (Y = 4–5.5 m), the model parameters are poorly constrained by the synthetic data.

As can be seen from Fig. 7b, which shows the results of inverting the synthetic dataset, the SP-NFC was correctly reconstructed. A high-resistivity anomaly (> 1500 Ωm) in the central region is distinguishable from a homogeneous background of 500 Ωm in all images. The anomaly, measuring 2.3 m in height and 2 m in width, begins at a depth of 1 m and extends to a depth of more than 2 m. The depth slices obtained from the 3D inversion model (Fig. 7b) show that the anomaly is generally symmetrical around the vertical joint dividing the left and right Chevron blocks.

### Inversion results of field data

**Fig. 8:**
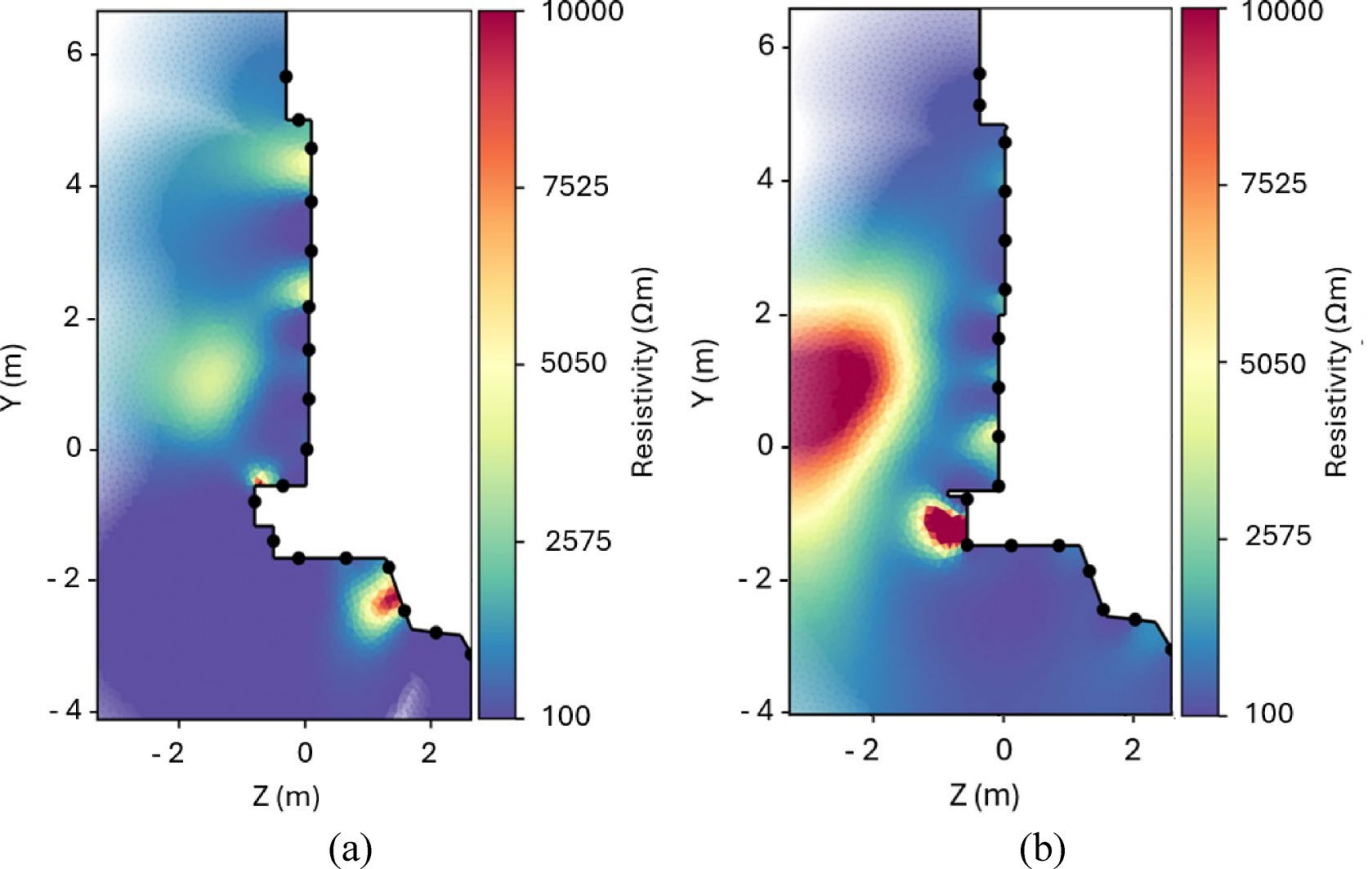
Inversion results for (**a**) 2D ERT line 8 at X = -0.65 m (left side of the Chevron) and (**b**) 2D ERT line 2 at X = 1 m (right side of the Chevron). The black points mark the position of the electrodes.

Before analyzing the 3D inversion model, the results were first interpreted using 2D ERT sections. Figure 8 presents examples of the 2D inversion results for two profiles: line 2 (X = 1 m) and line 8 (X = − 0.65 m), with coverage information visualized using alpha shading. In the figures, inverted resistivity values are displayed without shading in areas where the measured ERT data provide sufficient coverage, while regions with low coverage appear faded.

As indicated on the survey site map (Fig. 2b), these lines are located on the right and left sides of the Chevron, respectively. For line 2 at X = 1 m (Fig. 8b), the RMS error of 4.6% aligns well with the relative input error of 4% for the inversion. The coverage is satisfactory to a depth of 2 m, except near the top of the profile, where it decreases rapidly. In the inverted section (Fig. 8b), a high-resistivity anomaly, approximately 2 m in height, is visible in the centre of the Chevron. This anomaly shows a strong resistivity contrast against a moderately resistive background. It starts at a depth of around 1 m and extends deep into the pyramid, reaching depths of up to 3 m. Figure 8a shows the 2D inversion model for line 8 (X = − 0.65 m), measured on the left side of the Chevron. The obtained model has a higher RMS error of 6.2% compared to line 2. The inversion results reveal a highly resistive anomaly that is smaller in size than the one detected on line 2. The depth extent of the anomaly is difficult to determine due to insufficient coverage beyond 2 m. It is worth noting that near-surface anomalies appear at the joints between individual stone blocks in both cases.

Consequently, both the size and resistivity values of the anomaly vary between profiles. In several cases, the 2D inversion models accurately reproduced the main features of the SP-NFC, including its approximate size and location, as well as the pronounced resistivity contrast with the surrounding limestone, characteristic of an air-filled void. However, some profiles located on the left side of the Chevron showed a less prominent anomaly, appearing smaller in size.

**Fig. 9:**
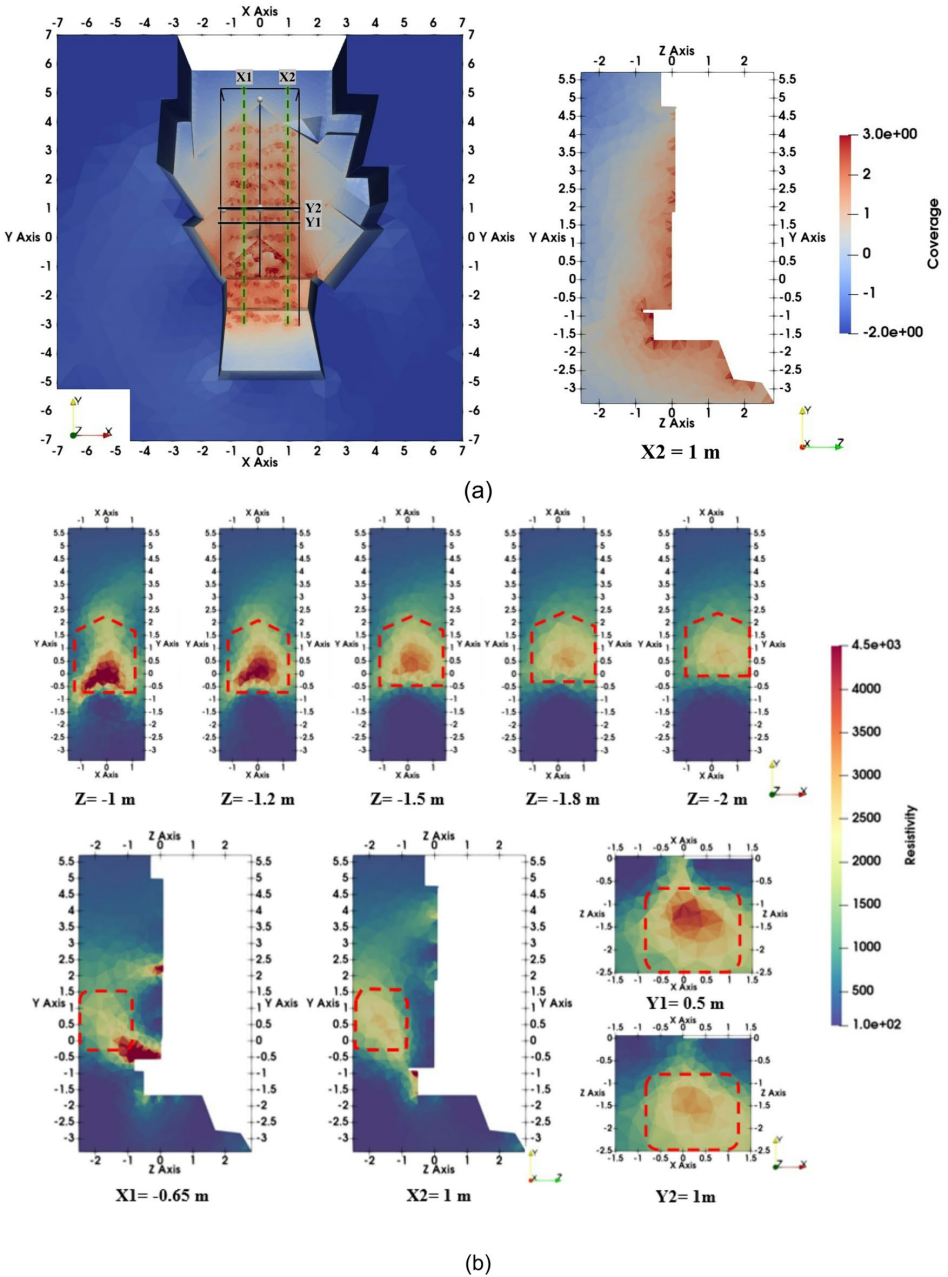
3D inversion results of the field data: (**a**) coverage of the entire model at the surface with the locations of the X and Y slices as well as the limits of the cropped area (black box) used for the inversion results (left) and coverage of slice X2 = 1 m (right); and (**b**) X-, Y-, and Z-slices of the inverted resistivity distribution.

The results of the 3D ERT inversion of the field data are presented in Fig. 9. The coverage distribution at X2 = 1 m (Fig. 9a) shows maximum values within the uppermost ~ 1 m, which is the region most strongly influencing the inversion results. Although the coverage gradually decreases with depth, it remains satisfactory down to approximately 2 m. The deeper parts of the model (below Z = − 2 m) and the region corresponding to the top of the Chevron (Y = 3.5–5.5 m) are not effectively covered by the acquisition geometry. The inversion algorithm converged with an RMS error of 16.19% after five iterations. The possible factors contributing to the relatively high RMS error will be discussed in Sect. 4.

Sections obtained from the 3D model at depths of 1.0 m, 1.2 m, 1.5 m, 1.8 m, and 2.0 m (Z-slices) are shown in Fig. 9b. A high-resistivity anomaly (> 4500 Ωm) in the central part of the study area is clearly visible at all depths. In the Z-slices, the anomaly has maximum dimensions of about 2.5 m by 2.5 m. Its greatest height is found in the central part of the study area. As can be seen from the model sections obtained in the vertical (parallel to the Y axis) and horizontal (parallel to the X axis) directions, the anomaly begins at a distance of around 1 m from the Chevron surface and extends to a depth of at least 2 m, which is close to the limit of the depth of investigation. As depth increases, the anomaly’s position shifts slightly upward, and due to decreased resolution, its resistivity contrast becomes less pronounced. Background resistivity values range from low (80–300 Ωm) to moderate (300–1500 Ωm).

## Discussion

Sections obtained from the 3D ERT model indicate that the corridor is approximately 2.5 m high and 2.5 m wide, extending along the Y-axis from − 0.2 m to 2.3 m, and along the X-axis from − 1 m to 1.5 m. The background resistivity values (300 to 1500 Ωm) are generally comparable to those expected for limestone, exhibiting variations likely related to the degree of weathering. In the near-surface zone, within the first meter, resistivity values are typically low (80 to 300 Ωm), perhaps explained by the weathered limestone.

**Fig. 10:**
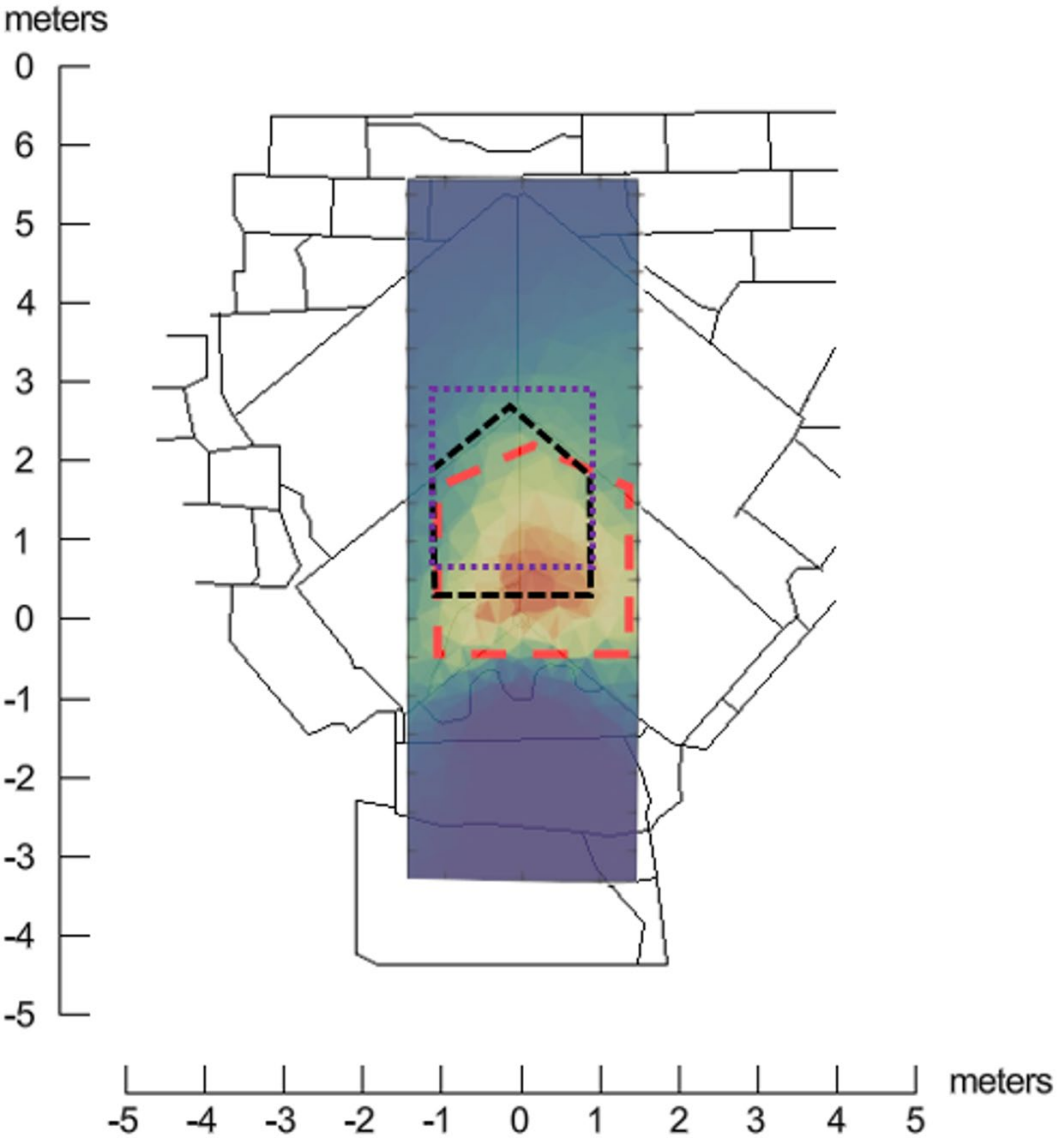
Sketch of the Chevron area located on the North Face of the Great Pyramid, overlaid with the ERT depth slice at Z = − 1.5 m (shown semi-transparent). The violet and black dashed lines indicate the estimated cross-sections of the SP-NFC obtained from muon radiography^5^ and GPR measurements^7^, respectively. The red dashed line outlines the approximate position of the SP-NFC derived from the ERT inversion results.

By comparing the ERT results (red dashed line in Fig. 10) with previously published studies of the SP-NFC, the accuracy of the estimated corridor position and dimensions can be evaluated. In particular, the most recent high-sensitivity muon radiography measurements^5^ provide refined information on the corridor’s location and geometry (violet dashed line in Fig. 10). In this study, the SP-NFC is reported to be 2.02 ± 0.06 m wide, 2.18 ± 0.17 m high, and 9.06 ± 0.07 m long, with its center along the Z-axis located at 2.0 ± 0.5 m. Concurrently, Elkarmoty and Rupfle, et al. (2023) reported the results of GPR surveys conducted with dual-frequency 200/600 MHz and 400 MHz antennas, along with UST measurements acquired on a high-resolution grid using two multi-channel ultrasonic pulse-echo systems operating at a frequency of 25 kHz. These data enabled a detailed reconstruction of the corridor’s cross-sectional geometry (black dashed line in Fig. 10). Specifically, the 400 MHz GPR radargram revealed the SP-NFC within the X-interval − 1.00 m to 1.00 m and Y-interval 0.30 m to 2.30 m in the local coordinate system of the Chevron area^7^. Videoendoscopic observations further confirmed these dimensions.

Figure 10 reveals slight differences in the SP-NFC’s estimated dimensions and position obtained from different methods. In particular, the ERT data overestimate the anomaly’s height and width by approximately 0.5 m each due to resolution limitations. In addition, in the depth sections of the ERT model between Z = 1.5 m and Z = 2 m, the position of the corridor is estimated more accurately, whereas at shallower depths, the anomaly shifts downward by 0.4 m to 0.5 m. This effect is most likely caused by large joints and air gaps below the SP-NFC, which strongly impact the resistivity distribution near the surface.

Compared to the inversion results of the synthetic ERT data, the anomaly associated with the corridor had a more complex shape and was asymmetrical with respect to the center. These discrepancies can be attributed to the fact that the synthetic model does not fully reproduce the real conditions; for example, individual stone blocks and joints are undetailed. Other factors that can affect the estimate of the anomaly’s height include air gaps between stone blocks and variations in limestone properties across individual blocks. In particular, higher resistivity values in areas surrounding the corridor may indicate more porous or cracked limestone, which could explain the blurred contours of the SP-NFC anomaly and its overstated size.

**Fig. 11:**
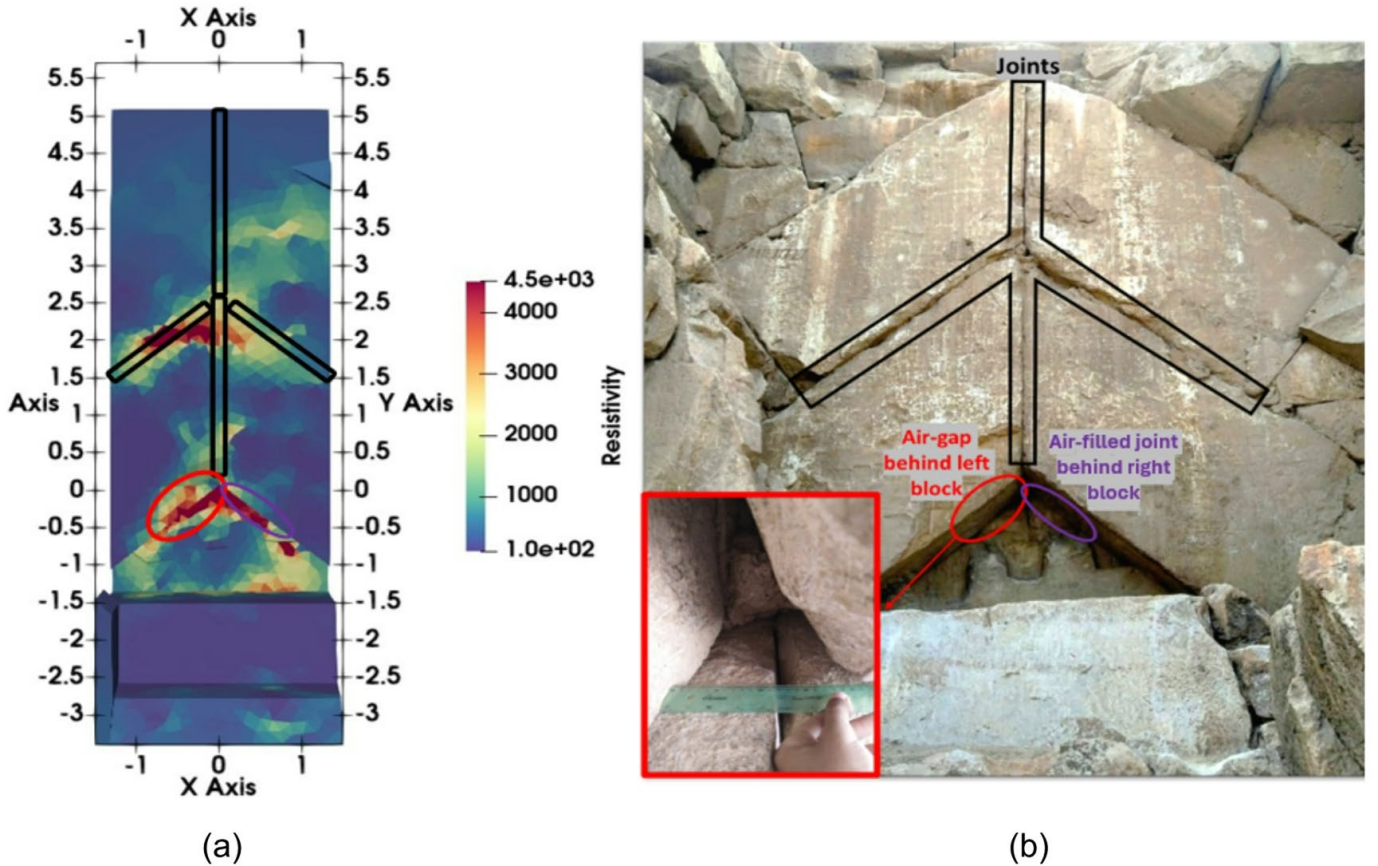
(**a**) Chevron surface (front view) from the 3D inverted model of the field data. High resistivity values correspond to the locations of partially opened, air-filled joints between separate blocks (shown in black), and to air gaps behind the left (in red) and right (in purple) blocks; (**b**) Photograph of the Chevron area with marked positions of joints and air gaps.

Another difference between the inversion of the synthetic and the field data is the higher resistivity contrast between the SP-NFC-related anomaly and the limestone in the latter. This is likely due to the open joints between the blocks directly in front of the SP-NFC (Fig. 11). Several joints between the stone blocks are visible on the X-, Y- and Z-slices of the 3D model, intersecting the main junctions of the Chevron blocks (Figs. 9b and 11a). Two wide joints are evident as high-resistivity anomalies spatially associated with the SP-NFC anomaly. The first originates from the air gap beneath the lower Chevron blocks, which allowed direct access to the corridor during videoendoscopic examination (Fig. 11b). The second is linked with the junction of the upper and lower Chevron blocks at a vertical distance of approximately 2.5 m. Around this location, in the upper right corner of the lower left Chevron block, GPR and UST data revealed high-amplitude reflections, likely caused by a discontinuity in the limestone^7^.

The presence of joints and the associated heterogeneity in the masonry’s physical properties may explain the high RMS error of the ERT inversion results. Over time, the binding material between individual blocks has degraded, leaving some joints either empty or filled with dust, sand, or clay^36^. Most joints are too small to be resolved in the ERT inversion. However, they affect the measurements to varying degrees, depending on the sensitivity of the measurements and the resistivity contrast between the joints and the building blocks. We suspect that this leads to a higher misfit (RMS error) and therefore want to investigate this problem more closely in the future.

The findings of this study should be interpreted in the context of the method’s limitations in resolution and depth of investigation. Based on the model coverage, resistivity variations are resolved only to a depth of roughly 2 m. Extending the length of the ERT line could help to increase the depth of investigation, but this was not possible in our case due to logistical constraints and the strong topography above the Chevron blocks.

Although the data were inverted on a fine mesh using a robust L1-norm approach with low regularization (λ = 1) to better resolve the corridor anomaly boundaries, these parameter settings cannot compensate for the smoothing effects inherent in the inversion process. This leads to blurring of the corridor anomaly. As the comparison of the ERT results with the GPR and UST data shows, the deviations in the estimated position and dimensions of the SP-NFC were within a margin of error of approximately 0.5 m.

## Summary and conclusions

In this study, ERT data collection, modeling, and inversion procedures were adapted to the Chevron area of the Great Pyramid to investigate the applicability of the ERT method in detecting a hidden corridor inside the pyramid, known as the SP-NFC. The ERT data was acquired using special lightweight mesh electrodes and processed with a 3D inversion algorithm. To effectively perform 3D finite element modeling of the study area, an optimized 3D CAD model of the Chevron area was created, simplifying the complex geometry of its surface.

An approximate estimate of the corridor’s location and dimensions was obtained from a 3D inversion model that incorporated the topography of the Chevron and its surrounding area. The model revealed a high-resistivity anomaly in the Chevron’s central part (X = − 1 to 1.5 m; Y = − 0.2 to 2.3 m), beginning at a depth of approximately 1 m and extending for at least 2 m. This interpretation was validated by comparing it with the results of numerical 3D modeling that reproduces the SP-NFC in the Chevron area, based on its known size and position obtained from GPR and UST data. However, the corridor’s entire length could not be reconstructed due to the limited investigation depth.

The study demonstrated that large open joints between stone blocks significantly influence the results, an effect that must be considered when interpreting ERT data from masonry monuments. Future research will further investigate this issue through numerical modelling of different scenarios regarding the location, orientation, and width of air-filled joints.

Overall, the study’s findings demonstrate the effectiveness of ERT in detecting the SP-NFC in the Chevron area of the Great Pyramid. Despite some limitations that may arise under different real conditions, this approach can be helpful for 3D visualization and structural characterization of stone monuments with complex surface geometry.

## Data Availability

The data will be made available from the corresponding author upon reasonable request and with permission from the Egyptian Ministry of Tourism and Antiquities.
